# Characterization of a Surface-Active Protein Extracted from a Marine Strain of *Penicillium chrysogenum*

**DOI:** 10.3390/ijms20133242

**Published:** 2019-07-02

**Authors:** Paola Cicatiello, Ilaria Stanzione, Principia Dardano, Luca De Stefano, Leila Birolo, Addolorata De Chiaro, Daria Maria Monti, Ganna Petruk, Gerardino D’Errico, Paola Giardina

**Affiliations:** 1 Department of Chemical Sciences, University of Naples (Federico II), Via Cinthia, 80126 Naples, Italy; 2 Institute for Microelectronics and Microsystems, Unit of Naples-National Research Council, Via P. Castellino 111, 80127 Naples, Italy

**Keywords:** marine fungi, biosurfactant proteins, amyloid fibrils, emulsions

## Abstract

Marine microorganisms represent a reservoir of new promising secondary metabolites. Surface-active proteins with good emulsification activity can be isolated from fungal species that inhabit the marine environment and can be promising candidates for different biotechnological applications. In this study a novel surface-active protein, named Sap-*Pc*, was purified from a marine strain of *Penicillium chrysogenum.* The effect of salt concentration and temperature on protein production was analyzed, and a purification method was set up. The purified protein, identified as Pc13g06930, was annotated as a hypothetical protein. It was able to form emulsions, which were stable for at least one month, with an emulsification index comparable to that of other known surface-active proteins. The surface tension reduction was analyzed as function of protein concentration and a critical micellar concentration of 2 μM was determined. At neutral or alkaline pH, secondary structure changes were monitored over time, concurrently with the appearance of protein precipitation. Formation of amyloid-like fibrils of SAP*-Pc* was demonstrated by spectroscopic and microscopic analyses. Moreover, the effect of protein concentration, a parameter affecting kinetics of fibril formation, was investigated and an on-pathway involvement of micellar aggregates during the fibril formation process was suggested.

## 1. Introduction

Most of the emulsifiers currently used are synthetic; however, in the era of green technology, great interest is being given to surface-active biomolecules [1]. These compounds offer many advantages over their synthetic counterparts thanks to their biodegradable and environmentally friendly nature [2,3]. The constant research of efficient surface-active compounds, biosurfactants (BSs), and bioemulsifiers (BEs), with improved thermo-physical properties could make several industrial processes more sustainable. Indeed, these molecules find applications in cosmetics, pharmaceutics, food processes, and bioremediation [4,5,6]. In an oil polluted environment, these molecules play a specific role of binding to dispersed hydrocarbons and oils, preventing them from merging together, thus increasing their access and availability for biodegradation. These amphiphilic molecules mainly produced by microorganisms occur in nature as different kinds of compounds characterized by low molecular weight, i.e., glycolipids, lipopeptides, phospholipids, and fatty acids, or high molecular weight, i.e., amphipathic polysaccharides, proteins, lipopolysaccharides, lipoproteins or complex mixtures of these biopolymers [7]. 

According to Uzoigwe et al. [8], the terms BE and BS are not interchangeable, because they differ for physico–chemical properties and physiological roles. The low molecular weight compounds, known as BSs, have excellent surface activity, reduce the surface and interfacial tension between different phases, possess a low critical micelle concentration (cmc), and form stable emulsions. On the other hand, BEs are the high molecular weight compounds, which can efficiently emulsify two immiscible liquids even at low concentrations but are less effective at surface tension reduction. Therefore, they possess emulsifying activity, but not high surface activity. 

In this last decade, different habitats have been explored to isolate BE and BS compounds resistant to extreme conditions to replace their synthetic counterparts. Since marine microorganisms live in a stressful habitat, under cold, lightless, and high-pressure conditions or in association with other organisms, they represent a reservoir of promising secondary metabolites. Indeed, a wide variety of genera producing diverse types of surface-active compounds are associated with the marine environment [9,10,11]. Adaptive changes in secondary metabolite production by facultative marine microorganisms with respect to their terrestrial counterparts have been observed in response to environmental variations in pressure, temperature, and salinity [12]. Among the marine microorganisms, production of surface-active compounds from bacterial species are well explored, whereas little is known about their production from fungi [13,14]. In most of the studies, these biomolecules were only partially characterized, and their activities analyzed using the crude extracts rather than purified molecules [15,16]. Among them, few proteins were isolated and classified as BE. To date, most surface-active proteins known to be produced by fungi are the hydrophobins (HPBs) [17,18]. The intriguing features of these proteins rely on their amphiphilic nature, intrinsically related to their 3D structure. They self-assemble at the hydrophilic/hydrophobic interface, stabilizing air bubbles and water/oil emulsions [19,20,21]. The HPB family can be divided into two distinct classes: Class I HPBs self-assemble into very stable layers, forming amyloid-like fibrils, resistant to very harsh conditions (hot 2% sodium dodecyl sulphate); and Class II HPB aggregates are nonfibrillar and less stable, and can be more easily dissolved in detergent or organic solvents. 

A screening of marine fungi was previously carried out to isolate new HPBs or other surface-active proteins [22,23]. Twenty-three fungi were selected for their ability to produce foam during their growth in shaking culture. Extraction methods were set up to isolate secreted or cell wall associated HPBs, allowing the identification of six new putative HPBs. The protein produced by *Penicillium chrysogenum* MUT 5039 was endowed with the best emulsification capacity tested on a mixture of water and olive oil. Actually, marine isolates of *P. chrysogenum* were already proven to be good sources of new and interesting bioactive compounds [13]. Based on the stability of the amphiphilic layer it formed, the protein was predicted to be a member of the Class II HPB family [22]. This proteic BE was very promising from a biotechnological point of view and was herein characterized. At first, the attention was focused on the protein production yield and on its purification, then the protein was characterized and its surface activity analyzed. Unexpectedly, it was not an HPB but a previously unknown proteic BE, which forms amyloid-like fibrils.

## 2. Results

### 2.1. Purification and Identification of the Penicillium chrysogenum BE Protein

The strain *P. chrysogenum* MUT 5039 was previously selected as a good BE producer from a pool of marine fungi [22]. This is a facultative marine fungus, able to grow in the presence or absence of NaCl, suggesting that it may have been spilled into the sea and then adapted to the new salty environment [24,25]. In the previous work [22], the sodium dodecyl sulphate polyacrylamide gel electrophoresis (SDS–PAGE) analysis of the proteins extracted from the culture broth of this fungus after bubbling, showed only one main protein band. 

Herein, to select the optimal fungal growth conditions to obtain the protein, the strain was grown in liquid medium in the presence of 0%, 1.5%, and 3% NaCl at 20 °C and 28°C. The fungal growth was not significantly affected by salt concentrations or temperature; indeed, the same amount of mycelium was produced in all conditions (about 10 g of dry mycelia per liter of culture). On the other hand, the highest protein yield was obtained at 20°C supplying the medium with 1.5% NaCl, obtaining about 30 mg of protein per liter of culture (Figure S1). 

**Figure 1:**
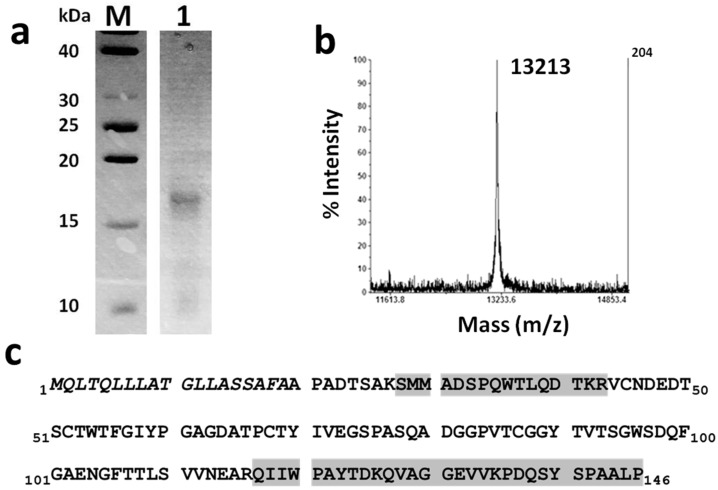
(**a**) SDS analysis of SAP*-Pc* in 60% ethanol solution after methanol chloroform treatment; (**b**) MALDI-TOF spectrum of SAP*-Pc* in 60% ethanol solution in linear mode; (**c**) sequence coverage of the primary structure of identified SAP*-Pc* highlighted in grey.

After air-bubbling of the culture broth, 80% of the initial protein amount was recovered in half of the original volume. However, samples before and after bubbling, analyzed by SDS–PAGE, showed smeared bands (Figure S2). Similar results were obtained even after trichloroacetic acid (TCA) precipitation and dissolution of the concentrated sample in 60% ethanol. The presence of lipid contaminants proven by Fourier-transform infrared spectroscopy (FTIR) and thin-layer chromatography (TLC) analysis, was significantly reduced after methanol chloroform extraction (Figure S3). Then a sharp SDS–PAGE band of the protein, dissolved in 60% ethanol, was obtained (Figure 1a, lane 1). This band was analyzed by a proteomic approach with LC–MSMS analysis, leading to the identification of the protein Pc13g06930 (ID NCBIr 255936199), with five peptides, for a total sequence coverage of 31% (Figure 1c, Table S1). The molecular mass of the protein, named SAP-Pc, was 13,213 m/z, as estimated by MALDI-TOF (Figure 1b), whereas the expected mass of Pc13g06930, without the putative signal peptide, was 13,313 m/z. This 100 Da difference could be due to aminoacidic substitutions or post-translational modifications occurring in this strain.

Pc13g06930, annotated as hypothetical protein, is similar to many other uncharacterized proteins found in different fungal species, whose genomes are known. The hydrophobicity plot in Figure S4 shows that the first half of the sequence is more hydrophilic than the second half.

### 2.2. Protein Characterization as Biosurfactant

At first, the concentration of SAP*-Pc* in aqueous buffers at three different pHs (4, 7, and 9) was verified to be the same as in 60% ethanol solution (100 µg/mL).

**Figure 2:**
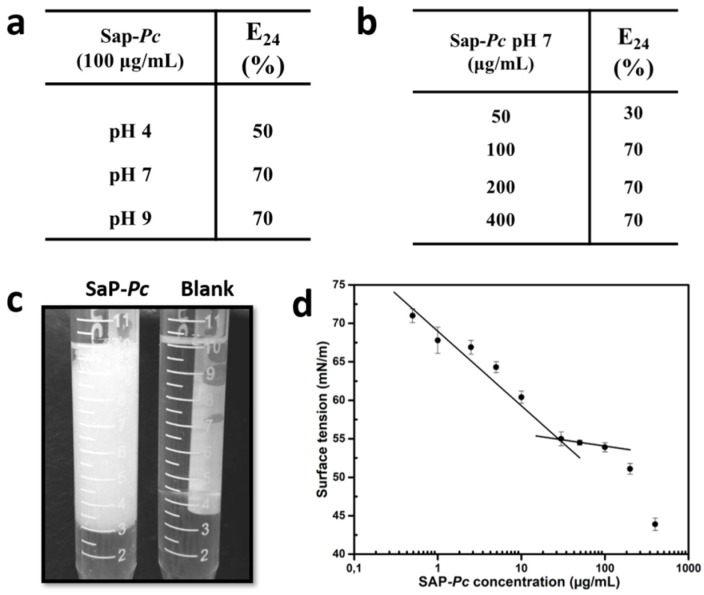
(**a**) Table of E_24_ values of 100 µg/mL of SAP*-Pc* at different pHs in the presence of Dectol; (**b**) table of E_24_ values of SAP*-Pc* dissolved at pH 7 at different concentrations in the presence of Dectol. All results are averages from three replicate experiments and the standard deviation is less than 10%. (**c**) Emulsion of 100 µg/mL SAP*-Pc* in 10 mM phosphate buffer at pH 7 (4 mL) mixed to 6 mL of Dectol after 24 h, in comparison to the mixture of buffer and Dectol, in the absence of the protein. (**d**) graph of surface tension of SAP*-Pc* in 10 mM phosphate buffer at pH 7 as function of protein concentration.

The SAP*-Pc* emulsification ability was tested in the presence of a “model oil”, Dectol [26]. The emulsification index E_24_ of samples dissolved at 100 µg/mL at different pHs (4, 7, and 9) was measured, as reported in Figure 2a. The best performance as emulsifier was obtained at both neutral and alkaline conditions, showing an E_24_ of about 70%. These emulsions remained stable for at least one month. On the contrary, the E_24_ in acidic conditions was lower (50%) and the emulsion less stable during the time. The E_24_ at different SAP-*Pc* concentrations was evaluated at pH 7 (Figure 2b). Doubling the protein concentration from 50 to 100 µg/mL, the E_24_ increased from 30 to 70%. After that, the E_24_ value was almost constant up to the maximum concentration used (400 µg/mL).

Surface tension of solutions of SAP*-Pc* dissolved at different concentrations in phosphate buffer with pH 7, was measured (Figure 2d). It must be noted that the lowest value at each protein concentration was reached slowly, as is typical for proteins [27,28], and at least one hour was needed to reach the final equilibrium value. When SAP*-Pc* concentration increased from 0.5 to 30 µg/mL, the surface tension was gradually reduced from 72 mN/m of the aqueous buffer to 55 mN/m; then the value remained almost constant from 30 to 100 μg/mL. Therefore, a cmc of 28 µg/mL (2 μM) was calculated as the point of intersection between two trend lines. At protein concentrations higher than about 100 μg/mL, a steep decline of the surface tension indicated the occurrence of another aggregation phenomenon.

### 2.3. SAP-Pc Aggregation

The solution of SAP-*Pc* in 60% ethanol remained stable for several months at room temperature. On the other hand, when the protein was stored at 100 µg/mL in aqueous buffers at different pHs, a concentration reduction (60–70%) was observed after about four days at pH 7 and 9, while no variation was detected at acidic pH. Hence further analyses were performed to investigate SAP-*Pc* aggregation processes.

**Figure 3:**
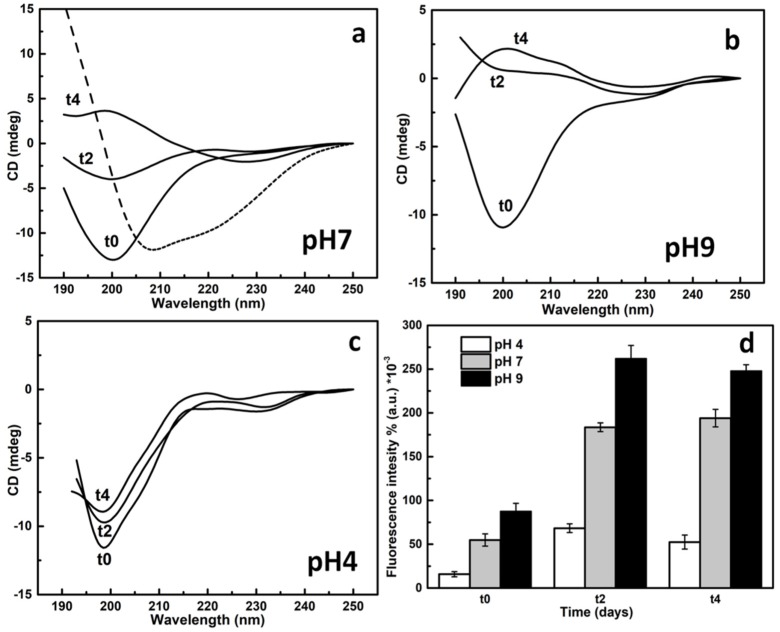
Circular dichroism (CD) spectra of SAP*-Pc* (100 μg/mL) dissolved in aqueous buffers at pH 7 (**a**), pH 9 (**b**), and pH 4 (**c**), just after dissolution (t0) and after 2 and 4 days. The dotted line in panel a corresponds to the spectrum of the protein in 60% ethanol; (**d**) ThT assay: fluorescence intensity of the same samples in the presence of 30 μM ThT.

The protein dissolved in the aqueous buffers showed a random coil structure when analyzed by far UV-circular dichroism (CD), with a minimum at 200 nm at all pHs used (4, 7, and 9), whereas it was more structured in 60% ethanol solution, as expected (Figure 3). The CD spectra of the protein incubated at room temperature in the three buffers were recorded. At neutral and alkaline conditions, the protein showed significant conformational changes during that time (Figure 3a,b). On the contrary, the spectra of SAP*-Pc* in acidic conditions, or in 60% EtOH, appeared unchanged over time (Figure 3c).

**Figure 4:**
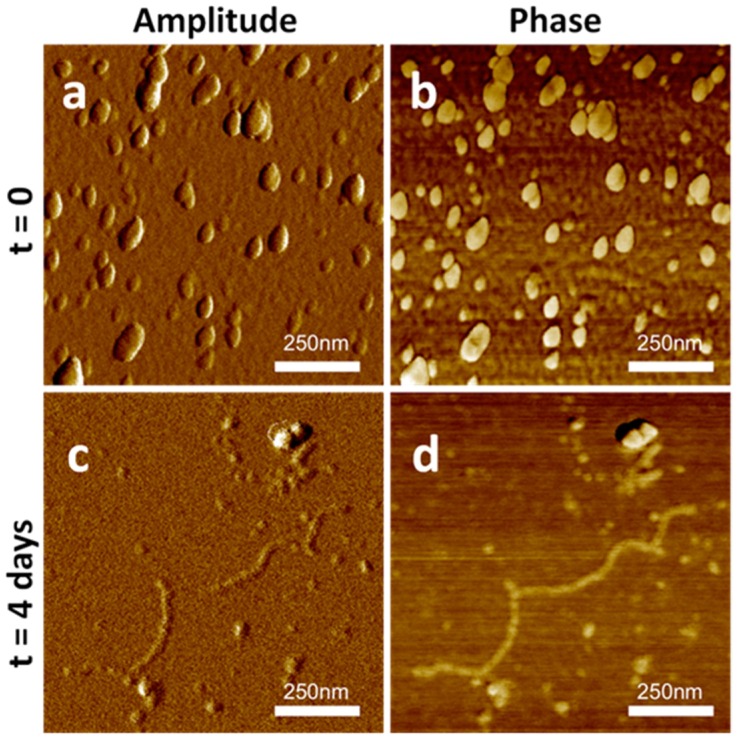
AFM imaging of 100 μg/mL SAP*-Pc* in 10 mM phosphate buffer pH 7 after washing: Non-contact mode (NCM) amplitude (left column) and phase (right column) at t_0_ (**a**,**b**) and after 4 days (**c**,**d**).

Taking into consideration that these structural changes led to a higher contribution of β structures (e.g., see the spectrum after 4 days at pH 7), the formation of amyloid-like fibrils can be envisaged. The fluorescent dye thioflavin-T (ThT), typically used to detect the presence of amyloid fibrils, was used to verify their formation. A significant increase of the ThT fluorescence intensity was observed at neutral and alkaline pHs during a period of one week (Figure 3d). To confirm the formation of amyloid-like structures, atomic force microscopy (AFM) analysis of SAP*-Pc* was performed, just after dissolution in aqueous buffer at pH7 (t0), and after 4 days (Figure 4). At t0, ellipsoidal protein aggregates were observed (54 ± 30 nm), whereas fibrils were detected in samples 4 days after dissolution. Their length was more than 1 µm, the medium width was 30nm, and their shape looked like an alignment of aggregates.

### 2.4. Effect of Protein Concentration on SAP-Pc Aggregation

**Figure 5:**
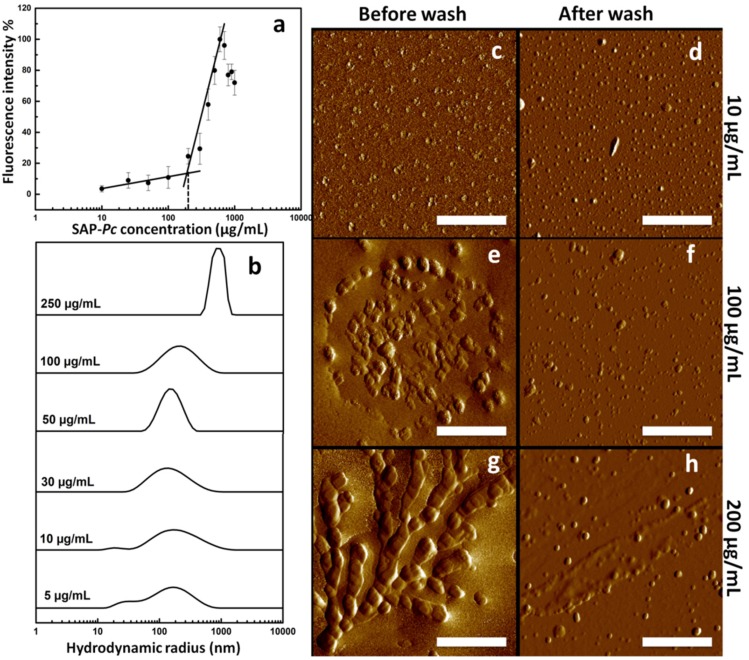
(**a**) Fluorescence intensity of SAP*-Pc* dissolved in 10 mM phosphate buffer at pH 7 at different concentrations in the presence of 30 μM ThT; (**b**) the averaged-intensity of hydrodynamic size distribution of SAP*-Pc* dissolved in 10 mM phosphate buffer at pH 7 at different concentrations. (**c**–**g**) AFM imaging of 10 (**c**,**d**), 100 (**e**,**f**), and 200 (**g**,**h**) μg/mL, respectively from top to bottom, SAP-*Pc* in 10 mM phosphate buffer at pH 7. Non-contact mode (NCM) amplitude images of casted samples before (left column) and after washing (right column) (scale bar is 1 μm).

Protein concentration is an important parameter affecting self-assembling processes [29,30]. Indeed, the ThT fluorescence intensity remarkably increased at SAP*-Pc* concentrations ranging from 200 to 600 µg/mL at pH 7 (Figure 5a), while its decrease at higher concentrations was probably due to protein precipitation. Therefore, a concentration parameter based on ThT assays can be established and can be named critical aggregation concentration (cac), according to other authors [31,32]. Its value was 191 µg/mL (14 µM), calculated as the point of intersection between two trend lines.

Formation of SAP*-Pc* aggregates at different protein concentrations (pH 7) was also analyzed by dynamic light scattering (DLS). At protein concentrations ranging from 5 to 10 µg/mL, a population with a hydrodynamic radius of about 20 ± 9 nm was observed together to a population of 140 ± 20 nm, which is very large for a 13 kDa protein, (i.e., lysozyme: M_W_ = 14.5 kDa, R_H_ = 1.9 nm [33]) thus demonstrating the presence of aggregates even at these low concentrations. The population of smaller aggregates appeared more evident in the graph of volume-averaged size distribution (Figure S5). From 10 to 100 µg/mL the main peak remained centered at 140 ± 20 nm, while at 250 µg/mL a R_H_ value of 830 ± 30 nm was reached (Figure 5b,c). At 400 µg/mL the sample appeared too heterogeneous and poly-dispersed to obtain reliable measurements.

AFM analysis of samples at 10, 100, and 200 µg/mL were in line with these results. Statistics, as shown in Figure 5d,e,g, gave an average radius of 50 ± 30 nm, 110 ± 70 nm, and 150 ± 90 nm, for 10, 100, and 200 µg/mL concentrations, respectively. Furthermore, very long alignments of aggregates forming fibrils were detected in the more concentrated sample. However, these long fibrils tended to leave the mica surface when washed.

### 2.5. Toxicity Analysis

As a preliminary approach in view of a potential use in the cosmetics or medical field, SAP*-Pc* biocompatibility was investigated on human immortalized keratinocytes, which represent the outer part of the skin and are considered as guard cells for the human body. Cells were incubated in the presence of increasing amounts of SAP*-Pc* for 24 and 48 h and cell viability was determined. As shown in Figure S6, SAP*-Pc* induced 20–25% of toxicity after 24 h incubation at 50 and 100 µg/mL, and 20–30% of toxicity after 48 h incubation.

## 3. Discussion

The protein SAP-*Pc* was purified from the culture broth of a facultative marine strain of *P. chrysogenum*, whose selection was based on its ability to form foam during growth. The purification procedure used was set up to isolate Class II HPBs, known fungal surface-active proteins, secreted in culture media [22]. Nevertheless, the principal protein secreted by this fungus, under the stressful conditions of the marine habitat, was not HPB but an unknown protein, which we named SAP-*Pc*. The emulsification ability of SAP-*Pc* is similar to that reported for HFBII, a Class II HPB [26] in the same conditions and the emulsions obtained in the presence of SAP-*Pc* are very stable [22]. Moreover, its low toxicity suggests potential applications in the cosmetics and biomedical fields, considering also its low cost in terms of culture broth and purification procedure.

Oligomeric assemblies formed by amphiphilic proteins can be considered as “micelle(s)”, with properties consistent with those of well characterized micelle-forming substances [34]. Taking into consideration the first two regions of the plot of surface tension vs. log of surfactant concentration, a cmc can be determined, like that of any surfactant. The surface tension decreased when the cmc was small, as for other BEs [8]. However, it was clear from the same plot that other aggregation phenomena occurred at higher concentrations (more than 200 μg/mL).

Indeed, formation of amyloid like fibrils of SAP-*Pc* was demonstrated at neutral and alkaline pHs, and as a function of time and protein concentration, parameters that generally affect protein aggregation phenomena. On the other hand, the protein remains soluble at pH 4, adopting random coil conformations. Aggregates of ellipsoidal shapes were observed by AFM and detected by DLS already at 10 µg/mL. Four days after dissolution, or at a protein concentration of 200 µg/mL, fibrils were observed by AFM and a precipitate was perceptible to the naked eye. Formation of amyloid-like fibrils at pH 7 was observed by all the techniques used. It is worth noting the typical morphology of these fibrils: they look like a “pearl neck-lace”, which can be originated by an array of interacting aggregates. According to the graph of the ThT fluorescence assays vs log of protein concentration, and DLS analysis, a cac can be determined. Hence, the notable decrease of the surface tension observed at concentrations higher than cac can be related to the fibril formation and to their tendency to reside at the interface. Indeed, the behavior of amyloid peptides has often been compared to that of surfactants [31,35]. The concentration dependence of the degree of aggregation is typical for spontaneous cooperative aggregation processes, such as the self-assembly of surfactants into micelles. Usually in these processes, monomers are present in solution at low concentrations, while aggregates form when the concentration exceeds a fixed value.

In the case of SAP-*Pc*, two different phenomena have to be considered, being that the cmc and cac parameters were noticeably different (28 µg/mL cmc,191 µg/mL cac). The presence of aggregates in the concentration range between 30 and 200 µg/mL, with hydrodynamic radius centered at 140 nm, was shown. Much larger aggregates, at and above 200 µg/mL, should correspond to amyloid-like fibril formation, as verified by AFM analysis.

According to Dear et al. [36], proteins can form globular oligomers, micelle-like structures, under appropriate conditions through hydrophobic bonding, because of their amphiphilic character. These morphologies can play the role of intermediates on the way to form the more structured β-sheet-containing species, the amyloid fibrils.

Even in the case of SAP-*Pc*, micelles should be on-pathway with respect to fibril formation and evolve towards fibrillar aggregates, which are formed overtime or more quickly at higher protein concentration. Indeed, DLS analysis in Figure S7 showed the dependence of the lag time to aggregation upon the protein concentration. A higher SAP-*Pc* concentration results in more rapid nucleus formation and reduction of the lag-phase of amyloid fibril formation, thus again indicating that the micelle-like aggregates are intermediates of the fibrillization process.

## 4. Materials and Methods

### 4.1. Culture Conditions and Protein Extraction

The fungal strain *Penicillium chrysogenum* MUT 5039 was provided by the Mycotheca Universitatis Taurinensis. The mycelium was maintained at 4 °C through periodic transfer on XNST30 (malt extract 3 g/L; yeast extract 3 g/L; NaCl 30 g/L; 10 g/L glucose; and 5 g/L peptone) agar plates. Mycelia were inoculated in 1 L flasks containing 500 mL of WM (10 g/L glucose; 2 g/L peptone; 1 g/L (NH_4_)_2_SO_4_; 0.5 g/L MgSO_4_·7H_2_O; 0.875 g/L KH_2_PO_4_; 0.125 g/L K_2_HPO_4_; 0.1 g/L CaCl_2_·2H_2_O; 0.05 g/L MnCl_2_; 0.001 g/L FeSO_4_; different amounts of NaCl, 0 or 15 or 30 g/L), grown at 20 or 28 °C in shaken mode (180 rpm). After 5 days of fungal growth, the culture broth was separated from the mycelium by filtration through Whatman paper and agitated in a Waring blender to produce foam. Next, the foam was recovered and treated with 20% trichloroacetic acid (TCA), incubated overnight at 4 °C, and centrifuged for 1h at 3300× *g*. The precipitate was collected, dissolved in 60% ethanol aqueous solution, sonicated in Elmasonic S30 water bath sonicator (Elma Schmidbauer GmbH, Singen, Germany) for 20 min, and again centrifuged. The ethanol solution was used because HPBs are usually more soluble and stable in this condition. The raw extract was dried, and lipids were extracted in a mixture of methanol−chloroform 2:1 *v*/*v* (5 min in bath sonicator). After centrifugation, the protein pellet was dried and dissolved again in 60% ethanol. The protein concentration was evaluated using the PIERCE 660 nm protein assay (ThermoFischer Scientific, Waltham, MA, USA), with bovine serum albumin as the standard. The purity and the molecular weight of the extracted sample was evaluated by SDS–PAGE (15% acrylamide), stained by Coomassie brilliant blue.

### 4.2. Mass Spectrometry

MALDI mass spectra were recorded on a Sciex 5800 MALDI–TOF–TOF mass spectrometer (AB Sciex, Foster City, CA, USA). The analyte solutions were mixed with sinapinic acid (20 mg/mL in 70%acetonitrile, TFA 0.1% *v*/*v*) as the matrix, applied to the sample plate, and air dried. The spectrometer was used in the linear mode. The spectrum was calibrated externally.

SDS–PAGE analysis was performed to select the band of interest, which was then cut from the gel, destained by washes with 0.1 M NH_4_HCO_3_ pH 7.5 and acetonitrile, reduced for 45 min in 100 µL of 10 mM dithiothreitol, 0.1 M NH_4_HCO_3_, pH 7.5, and carboxyamidomethylated for 30 min in the dark by the addition of 100 µL of 55 mM iodoacetamide dissolved in the same buffer. Enzymatic digestion was performed and analyzed by LC–MSMS on a 6520 Accurate-Mass Q-TOF LC/MS system (Agilent Technologies, Palo Alto, CA, USA) equipped with a 1200 HPLC system and a chip cube (Agilent Technologies) as previously reported [37]. Peptide analysis was performed using data-dependent acquisition of one MS scan (mass range from 300 to 1800 m/z) followed by MS/MS scans of the five most abundant ions in each MS scan. The acquired MS/MS spectra were transformed in Mascot generic format (mgf) and used for protein identification in the unreviewed set of protein entries that are present in the NCBInr database for all fungi, with a licensed version of MASCOT software (http://www.matrixscience.com) version 2.4.0, with additional search parameters. Ion score was −10 log(P), where P is the probability that the observed match is a random event. Individual ion scores >45 indicated identity or extensive homology (*p* < 0.05). Protein scores were derived from ion scores as a non-probabilistic basis for ranking protein hits (http://www.matrixscience.com/help/interpretation_help.html).

### 4.3. Emulsification Index

The protein in 60% ethanol was dried and re-dissolved in 10 mM phosphate buffer (pH 7), or 10 mM sodium acetate (pH 4), or 10 mM Tris HCl (pH 9). Then, it was mixed with Dectol (decane and toluene in 65:35 volume ratio), which was used as a ‘model oil’ [26]. In a typical experiment, Dectol (6 mL) was added to the biosurfactant solution (4 mL) in a graduated tube. Then, the mixture was homogenized in a vortex for 2 min at maximum speed at room temperature. After 24 h the emulsification index, E_24_, was determined, calculating the ratio between the height of emulsifying layer and the total height, multiplied by 100.

### 4.4. Spectroscopy Techniques

CD spectra were recorded on a Jasco J715 spectropolarimeter (Jasco Corporation, Cremella (LC), Italy) equipped with a Peltier thermostatic cell holder in a quartz cell (0.1 cm light path) from 190 to 250 nm. The temperature was kept at 20 °C, and the sample compartment was continuously flushed with nitrogen gas. The final spectra were obtained by averaging three scans, using a bandwidth of 1 nm, a step width of 0.5 nm, and a 4 s averaging per point.

Fluorescence spectra were recorded at 25 °C with a HORIBA Scientific Fluoromax-4 spectrofluorometer (Horiba Italia, Rome, Italy). Slits were set to 3 and 6 nm spectral band-passes in excitation and emission monochromators, respectively. ThT, to 30 μM final concentration, was added, the samples were excited at 435 nm, and the emission was monitored from 460 to 560 nm.

### 4.5. AFM

An XE-100 atomic force microscope (Park Systems, Suwon, Korea) was used for the imaging of fibrils. Surface imaging was obtained in non-contact mode using silicon/aluminum coated cantilevers (SSS-NCHR 10M; Park Systems) 125 μm long with a resonance frequency of 204 to 397 kHz nominal force constant of 42 N/m and a typical tip radius 2 nm (<5 nm max). Here we used a low tip radius probe to improve measurements of fibril widths. The scan frequency was typically 0.5 Hz per line. When necessary, the AFM images were processed by flattening, in order to remove the background slope, and the contrast and brightness were adjusted. As done in [38], for sample preparation, muscovite mica with a surface area of ~1 cm^2^ was used as the substratum. The mica was freshly cleaved using adhesive tape prior to each deposition in order to ensure its cleanliness. The dried samples were dissolved in 10 mM phosphate buffer, pH7. 2 μL aliquots of protein were deposited on the substrates and the samples were dried by evaporation at room temperature under a ventilated fume hood. For washed samples, two min after deposition, the surfaces were gently washed with deionized water. Finally, the samples were dried as described above.

### 4.6. Surface Tension Measurements

Measurements were performed by using the De Nouy ring method with a KSV Sigma 70 digital tensiometer (Dyne Testing Ltd., Newton House, Lichfield, UK). An automatic device was used to select the rising velocity of the platinum ring and to set the time between two consecutive measurements. Thorough attention must be paid in using the De Nouy ring method to deduce bulk properties, because the surfactant adsorption kinetics can influence the results [39]. In our experiments, we set the ring rising velocity low enough to reach the equilibrium between the air–solution interface and the bulk solution. Instrument accuracy was checked to be better than 0.10 mN·m^−1^ by measuring γ for 10 mM phosphate buffer, pH 7, before each session of measurements. At least two independent replicates of each sample at different protein concentrations were measured.

### 4.7. Dynamic Light Scattering

The size evaluation of each sample was performed by dynamic light scattering (DLS). A Zetasizer Nano ZSP instrument (Malvern Instruments, Malvern, UK) equipped with a He–Ne laser (633 nm, fixed scattering angle of 173°, room temperature 25 °C) was used. The protein samples were dissolved in 10 mM phosphate buffer pH 7 and filtered (0.22 μm) before each analysis.

### 4.8. Cytotoxicity Assay

Immortalized human keratinocyte cells (HaCaT) were from Innoprot (Derio, Bizkaia, Spain), and were cultured in Dulbecco’s modified eagle’s medium (Sigma-Aldrich, Milan, Italy), supplemented with 10% fetal bovine serum (HyClone, Logan, UT, USA), 2 mM L-glutamine, and antibiotics. Cells were grown in a 5% CO_2_ humidified atmosphere at 37 °C. To test the biocompatibility of the molecule, cells were seeded in 96-well plates at a density of 2.5 × 10^3^/well. Then, 24 h after seeding, cells were incubated with increasing amount of the molecule under test (from 10 to 100 μg/mL) for 24, 48, and 72 h. At the end of incubation, cell viability was assessed by the MTT assay as previously described [40]. Cell survival was expressed as a percentage of viable cells in the presence of the analyzed molecule, with respect to control cells. Control cells were represented by cells grown in the absence of the molecule and by cells supplemented with identical volumes of buffer (10 mM sodium phosphate, pH 7.4). Two-way ANOVA was performed as a statistical analysis.

## 5. Conclusions

The Sap-*Pc* is a proteic BE, herein identified, homologous to many other unidentified ascomycete proteins from *Fusarium, Gibberella, Aspergillus,* and *Thricoderma sp.* The surface activity and the emulsification ability of these fungal proteins should be analyzed to confirm the existence or otherwise of a new family of proteic BEs.

It is worth noting the high stability of the emulsions obtained in the presence of Sap-*Pc*, which remained unaltered even after one month. These results, together with the low toxicity and the good productivity of the protein, could predict its exploitation as a sustainable BE.

The aggregation process of Sap-*Pc* was also studied and the formation of amyloid-like fibrils in suitable conditions was observed, varying protein concentrations and time. Formation of micelles should be on-pathway with respect to fibril formation, which seems originated by an array of interacting aggregates.

## Supplementary Materials

Supplementary materials can be found at https://www.mdpi.com/1422-0067/20/13/3242/s1.

## Abbreviations

AFM	Atomic force microscopy
BE	Bioemulsifier
BS	Biosurfactant
cac	Critical aggregation concentration
CD	Circular dichroism
cmc	Critical micellar concentration
DLS	Dynamic light scattering
E24	Emulsification index
FTIR	Fourier transform infrared spectroscopy
HPB	Hydrophobin
LC–MSMS	Liquid chromatography tandem mass spectrometry
MALDI–TOF	Matrix assisted laser desorption ionization–time of flight
SDS–PAGE	Sodium dodecyl sulphate polyacrylamide gel electrophoresis
TCA	Trichloroacetic acid
ThT	Thioflavin T
TLC	Thin layer chromatography
