# Supplementary material for: Characterization of a Surface-Active Protein Extracted from a Marine Strain of Penicillium chrysogenum
Source: Int J Mol Sci. 2019 Jul 2;20(13):3242. doi: 10.3390/ijms20133242 (PMC6651339; doi:10.3390/ijms20133242)
Supplement: Supplementary file 1 [file ijms-20-03242-s001.pdf]

Supplementary materials

Characterization of a surface-active protein extracted by a marine strain of *Penicillium chrysogenum*

Paola Cicatiello[1], Ilaria Stanzione[1], Principia Dardano[2], Luca De Stefano[2], Leila Birolo[1], Addolorata De Chiaro[1],Daria Maria Monti[1], Ganna Petruk[1],Gerardino D'Errico[1], Paola Giardina[1*]

[1] Department of Chemical Sciences, University of Naples «Federico II», Via Cinthia, 80126 Naples, Italy; giardina@unina.com

[2] Institute for Microelectronics and Microsystems, Unit of Naples-National Research Council, Via P. Castellino 111, 80127, Italy; e-mail@e-mail.com

[*] Correspondence: giardina@unina.it; Tel.: +39 081 674319

Thin-layer chromatography (TLC)

Silica gel on TLC aluminium foil was used as stationary phase for TLC realization, and a mixture of toluene-chloroform-acetone (7:2:1) was utilized as the mobile phase. The samples were dissolved in chloroform. Ten percent phosphomolybdic acid in ethanol was used as colour developer through dipping.

Fourier-transform infrared spectroscopy (FTIR)

20 µL of the sample in 60% ethanol was deposited on a hydrophilic polytetrafluoroethylene (PTFE) membrane and FTIR quantitation was performed by the Direct Detect® spectrometer, an infrared (IR)-based biomolecular quantitation system. This instrument enabled simultaneous protein quantitation and lipid analysis in the same sample.

Table S1: Mascot search results of LC-MS/MS data against NCBInr database showing identified proteins' score, number of peptides, and sequence coverage.

ID NCBInr (gi number)	Protein name	Score	Peptides Sequence	Score	Sequence coverage (%)
255936199	Pc13g06930	102	R. QIIW PAYTDK.Q	45	31
			K. QVAGGEVVKPDQSYSPAALP. -	27	
			K.SMM ADSPQWTLQDTK.R + Oxidation M	44	
			K.SMM ADSPQWTLQDTKR.V + 2Oxidation M	33	
			R.QIIWPAYTDKQVAGGEVVKPDQSYSPAALP.- + Gln->pyro-Glu (N-term Q)	20	

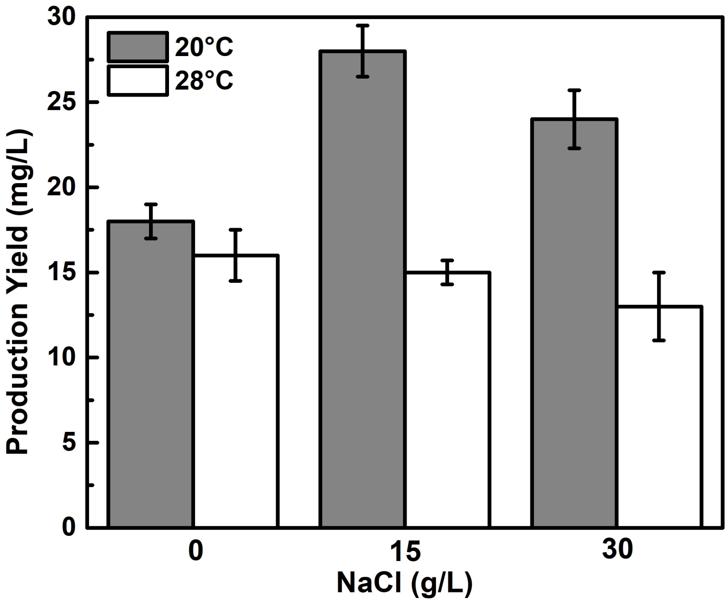

Figure S1: Protein production yield from *Penicilliumchrysogenum*at different temperatures and salt concentrations

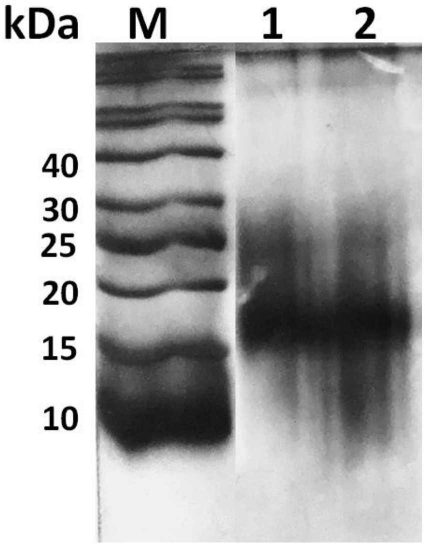

Figure S2: SDS-PAGE of filtrated culture broth **(1)** and air-bubbled culture broth **(2)**

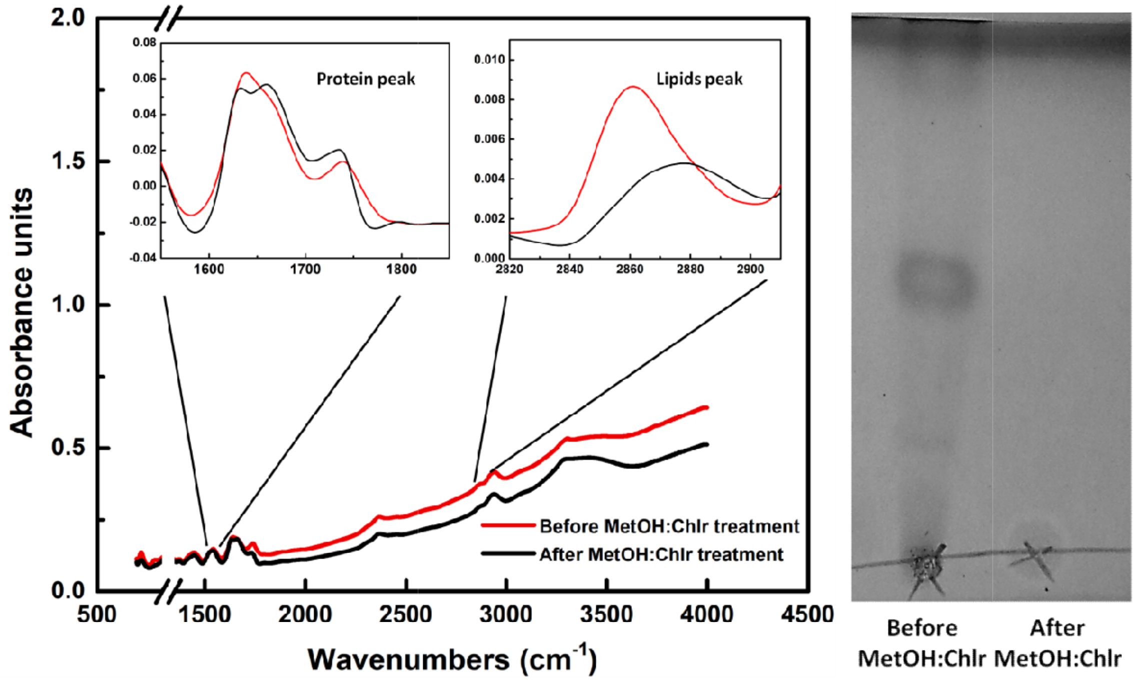

Figure S3:FTIR and TLC analysis of samples before and after the Methanol:Chloroform treatment to remove lipid contaminants. In the FTIR graph (left),the insets reported the main peaksrelated to proteinsandthe lipid peaks. The former remains unaltered after the treatment, while the lattersignificantly decreased.The TLC analysis (right)showed that thephosphomolybdic acid-stainedspot(related to lipids) was absentafter the treatment.

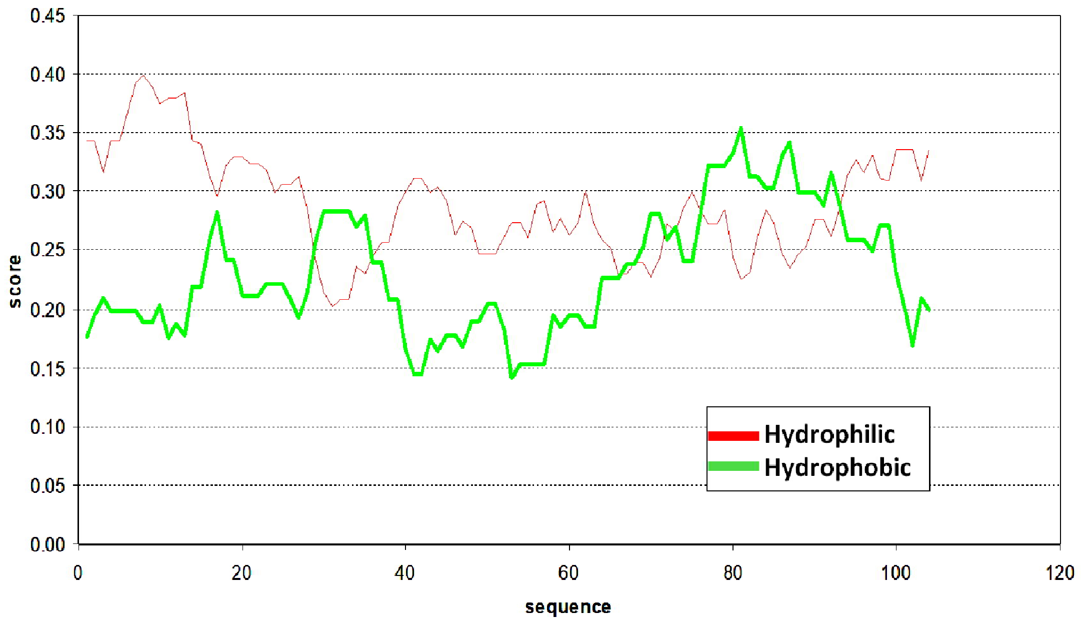

Figure S4: Hydrophobicity plot of Pc13g06930

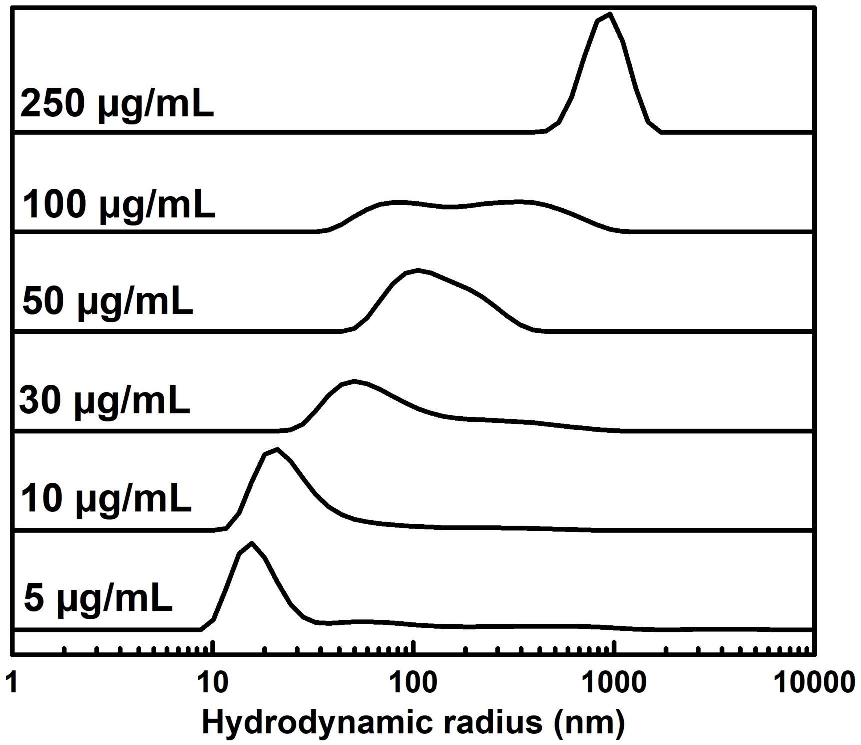

Figure S5:DLS analysis of Sap *Pc* at 100 µg/mL, in phosphate buffer pH 7, shown as volume-averaged size distribution.

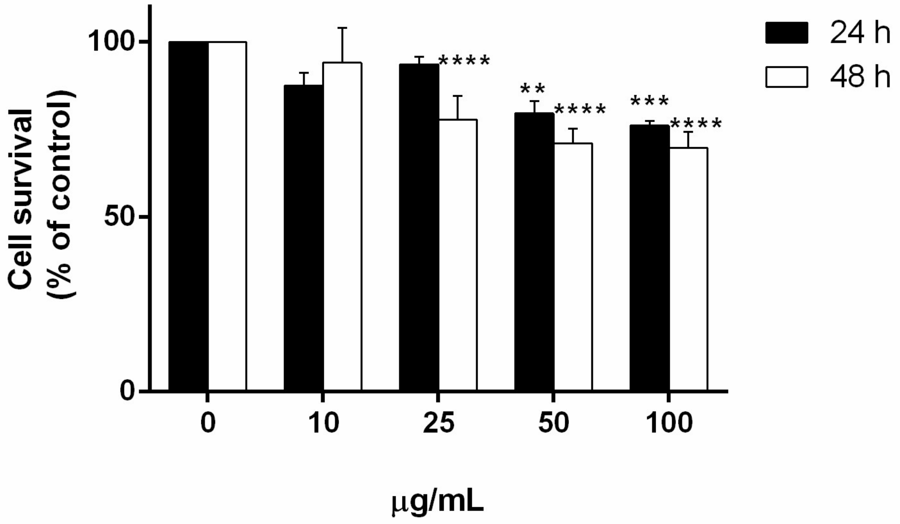

Figure S6: Effect of SAP-*Pc* on human immortalized keratinocytes. Dose-response plot of cells after 24-48 h incubation with increasing concentrations (10-100 µg mL^{-1}) of SAP-*Pc*. Cell viability was assessed by the MTT assay and expressed as described in Materials and Methods section. Values are given as means ± S.D. (n ≥ 3);** indicates p< 0.01, *** indicates p< 0.005, **** indicates p< 0.001, with respect to control cells.

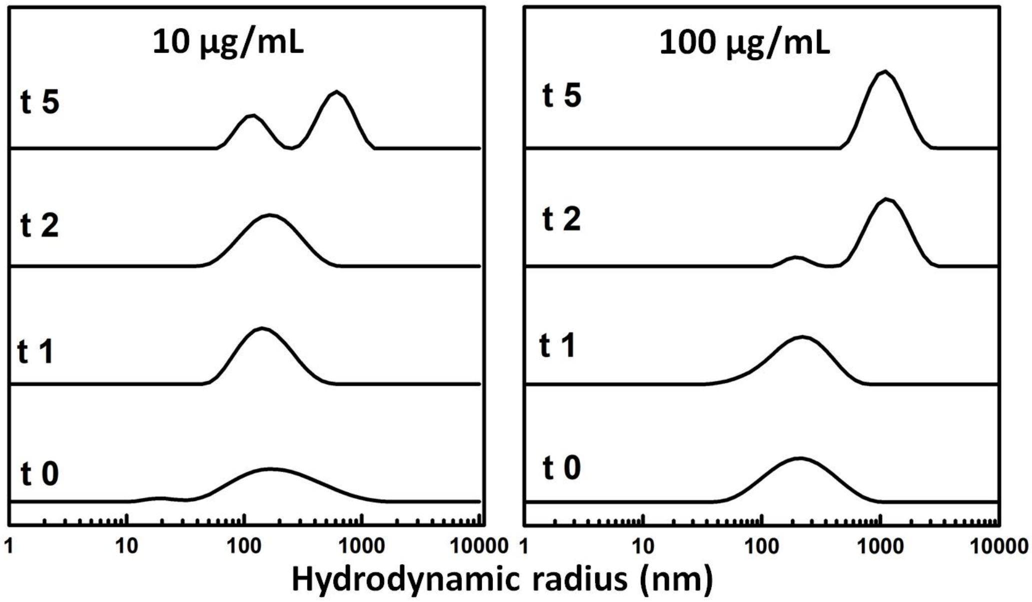

Figure S7:DLS analysis of Sap *Pc* at 10 and 100 µg/mL, in phosphate buffer pH 7, during time.
